# Left Ventricular Remodeling after Myocardial Infarction: From Physiopathology to Treatment

**DOI:** 10.3390/life12081111

**Published:** 2022-07-24

**Authors:** Sabina Andreea Leancă, Daniela Crișu, Antoniu Octavian Petriș, Irina Afrăsânie, Antonia Genes, Alexandru Dan Costache, Dan Nicolae Tesloianu, Irina Iuliana Costache

**Affiliations:** 1 Department of Cardiology, Emergency Clinical Hospital “Sf. Spiridon”, Bd. Independentei nr. 1, 700111 Iasi, Romania; sabinaandreea-leanca@email.umfiasi.ro (S.A.L.); antoniu.petris@umfiasi.ro (A.O.P.); irina-demsa@email.umfiasi.ro (I.A.); antonia.bobric@yahoo.com (A.G.); dan.tesloianu@umfiasi.ro (D.N.T.); irina.costache@umfiasi.ro (I.I.C.); 2 Department of Internal Medicine, “Grigore T. Popa” University of Medicine and Pharmacy, Str. University nr. 16, 700083 Iasi, Romania; adcostache@yahoo.com; 3 Department of Cardiovascular Rehabilitation, Clinical Rehabilitation Hospital, 700661 Iasi, Romania

**Keywords:** left ventricular remodeling, myocardial infarction, wall stress, inflammation, neurohormonal activation, heart failure

## Abstract

Myocardial infarction (MI) is the leading cause of death and morbidity worldwide, with an incidence relatively high in developed countries and rapidly growing in developing countries. The most common cause of MI is the rupture of an atherosclerotic plaque with subsequent thrombotic occlusion in the coronary circulation. This causes cardiomyocyte death and myocardial necrosis, with subsequent inflammation and fibrosis. Current therapies aim to restore coronary flow by thrombus dissolution with pharmaceutical treatment and/or intravascular stent implantation and to counteract neurohormonal activation. Despite these therapies, the injury caused by myocardial ischemia leads to left ventricular remodeling; this process involves changes in cardiac geometry, dimension and function and eventually progression to heart failure (HF). This review describes the pathophysiological mechanism that leads to cardiac remodeling and the therapeutic strategies with a role in slowing the progression of remodeling and improving cardiac structure and function.

## 1. Introduction

Postinfarct ventricular remodeling represents a prevailing cause of heart failure (HF), and it occurs in almost 30% of patients with a previous anterior myocardial infarction (MI) and in only approximately 17% of patients with non-anterior infarct [1].

Left ventricular (LV) adverse remodeling is a maladaptive process caused by cardiac injury characterized by morphological changes of LV structure and shape, with subsequent alteration of the cardiac function [2,3]. The term post-MI remodeling was first used in 1982 by Hockman and Buckey to describe the replacement of infarcted myocardium with scar tissue [4]. In 1990, Pfeffer and Braunwald published a review on ventricular remodeling after MI, showing that this process can affect the function of the ventricle and the prognosis of survival [5]. In the following years, the term cardiac remodeling was used to describe morphological and geometric changes in the LV after MI [6]. A consensus paper defines cardiac remodeling as a group of molecular, cellular and interstitial changes, which determine alterations in size, shape and function of the heart after cardiac injury [6].

In this narrative review, we have focused on the pathophysiological mechanisms and the assessment of cardiac remodeling post-myocardial infarction, as well as therapeutic approaches with proven or possible reverse remodeling effects. A comprehensive analysis of the current literature was made by searching Google Scholar and PubMed, using the keywords “cardiac remodeling”, “myocardial infarction”, “myocardial ischemia”, “cardiomyocyte metabolism”, “neurohormonal activation”. The results included original papers, prospective and retrospective studies, systematic reviews and meta-analysis, which underlined the pathophysiology of ventricular remodeling after myocardial infarction, as well as potential therapeutic strategies.

## 2. Pathophysiology of Adverse Remodeling

MI typically occurs consequently to the obstruction of epicardial coronary arteries. In an ischemic environment, cardiac myocytes develop an anaerobic metabolism with destabilization of the cell membrane and cell death through apoptosis, autophagy and necrosis [7,8,9]. Compared to other forms of myocardial injury, ischemic necrosis induces the death of millions of myocytes simultaneously, leading to an immune response and an influx of inflammatory cells into the infarcted area. Neutrophils and macrophages cause the destruction of the extracellular collagen matrix (ECM) and the expansion of the infarcted area, which consequently alter the ventricular shape, with thinning and dilatation of the infarcted myocardium. After reaching the maximum inflammatory response, fibroblasts are directed to the infarcted area where they create a new collagen matrix, and scar tissue is formed [3,10,11,12,13,14,15]. The inflammation may persist for a variable period of time after the ischemic event due to the continuous exposure of the myocardial wall to parietal stress. The compensatory activation of two major neurohormonal systems, the renin-angiotensin-aldosterone system (RAAS) and the sympathetic nervous system (SNS), in turn induces fibrosis and substantially intensifies the apoptotic changes. All of these processes lead to changes in the cardiac architecture and geometry, which are referred as adverse ventricular remodeling and are associated with a higher likelihood of HF and mortality [9,16].

Whereas ischemia caused by ST-segment elevation myocardial infarction (STEMI) in the absence of reperfusion therapy is more frequently associated with extensive scar formation and HF with reduced or mildly reduced ejection fraction (EF), in the case of non-ST-segment elevation myocardial infarction (NSTEMI), the adverse remodeling is rather associated with HF with preserved EF than with reduced EF. However, in terms of prognosis and mortality, there are no substantial differences between the two pathologies [16].

Furthermore, myocardial infarction can occur in the absence of obstructive coronary artery disease (MINOCA), and it is caused by various conditions, which create an imbalance between oxygen supply and demand, such as coronary vasospasm, coronary embolism, plaque disruption, microvascular dysfunction, tachyarrhythmias or arterial hypotension. Adverse remodeling occurs to a lower extent in this group of patients, as demonstrated by a study that showed that in more than half of the patients with a diagnosis of type 2 myocardial infarction, there was no imaging evidence of any functional consequences of myocardial infarction, such as a regional wall motion abnormality or scar formation. However, it is a known fact that these patients have a prognosis similar to those with an atherothrombotic MI, with a similar rate of future cardiovascular events [17].

### 2.1. The Sequence of Adverse Ventricular Remodeling

Ventricular remodeling following MI involves both the ischemic and the remote nonischemic myocardium, and it encompasses two stages. ***The early stage of remodeling*** occurs at the site of the infarct, a few hours from the acute coronary occlusion, continuing for nearly a week. In this phase, initial myocardial deformation by the persistent stretching of the myocytes activates stretch-induced signaling pathways mediated by a stretch sensor integrin, which will cause the pathological transformation of myocardial tissue with myocyte hypertrophy, followed by apoptosis and extracellular collagen matrix (ECM) changes with fibrosis. The cardiomyocyte lengthening and hypertrophy appear as an early compensatory mechanism for maintaining stroke volume (SV) following contractile tissue loss [6,11,13]. ***The second phase of remodeling***, also called the “late phase”, develops one month after the ischemic event, and it is characterized by potentially reversible structural and biochemical changes. It develops at a site distant from the infarcted area and it implies myocytes, which are still viable. Therefore, remote non-infarcted myocardial tissue becomes hypertrophied and dilated, as an adaptive response to the increased wall stress. This phase does not necessarily appear after every MI, and it is not always progressive [18,19,20,21].

### 2.2. Mechanisms of Adverse Remodeling

**Figure 1:**
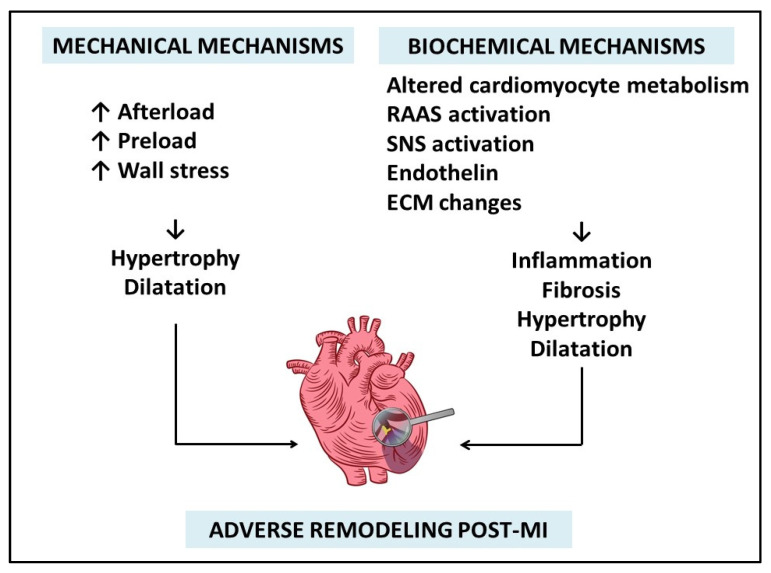
**Mechanisms of adverse ventricular remodeling after myocardial infarction.** RAAS: renin-angiotensin-aldosterone system; SNS: sympathetic nervous system; ECM: extracellular collagen matrix.

The main determinants of adverse ventricular remodeling post-myocardial infarction are represented by ***mechanical*** triggers and ***biochemical*** mechanisms, as described in Figure 1.

#### 2.2.1. Mechanical Pathophysiology

**Figure 2:**
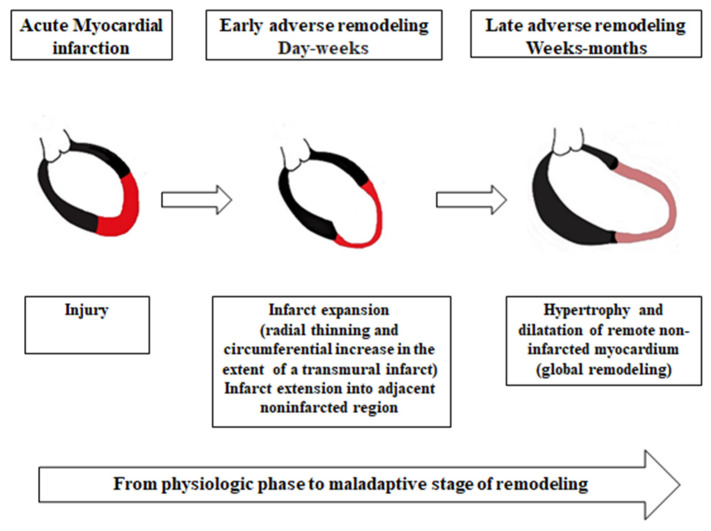
Mechanical mechanisms of adverse LV remodeling.

The most important mechanical factors, which determine the ventricular remodeling after MI, are changes in ***left ventricular geometry*** and ventricular ***wall stress*** (WS), as shown in Figure 2 [22]. The progression of cardiac remodeling is simple to understand, according to Laplace’s Law, which states that the LV WS is equal to the pressure in the chamber times the radius of the LV cavity, divided by the myocardial wall thickness. During the ***early phase*** of adverse remodeling, the infarcted segment is stretched due to a lack of counterbalance to the forces generated by the normally contracting myocardium. As a consequence, the increased wall tension from this level leads to a thinning of the infarcted wall and the expansion of the MI in the adjacent regions. Additionally, the stretched and dilated infarcted tissue increases the LV volume with a combined volume and pressure overload on the non-infarcted zones. During the ***late phase*** of remodeling, to maintain a normal stroke volume with a reduced number of properly functioning myocardial segments, in conditions of increased workload, the healthy cardiomyocytes will lengthen and progressively hypertrophy [23]. Finally, overstretching will cause the loss of the compensatory Frank–Starling mechanism and eventually lead to LV dilatation [20,22,24,25]. LV dilatation further increases WS, which in turn will accentuate chamber dilation and wall thinning, creating a vicious cycle [19,23,26].

As a consequence of the adverse remodeling process, the left ventricle loses its ellipsoid shape and becomes more spherical, with increased end-diastolic and end-systolic volumes. The relaxation, as well as the radial, longitudinal contraction and torsion movement, is depressed, leading to diastolic and systolic dysfunction. The distorted left ventricle geometry causes secondary mitral regurgitation through tethering mechanism, which leads to increased intraventricular pressures and further promotes the adverse remodeling process [13].

#### 2.2.2. Biochemical Pathophysiology

Adverse ventricular remodeling following myocardial infarction is the result of altered cardiomyocyte metabolism due to ischemic conditions, as well as the deleterious effects of several activated neurohormonal systems and molecular mechanisms.

##### Cardiomyocyte Metabolism

In the normally working myocardium, the energy required for myocyte contraction and relaxation results from the utilization of substrates, such as free fatty acids and carbohydrates by β-oxidation and glycolysis. The resulting metabolites will enter the tricarboxylic acid cycle (Krebbs cycle) in the mitochondria where energy will be produced by oxidative phosphorylation and stored in the form of high energy phosphates, mainly adenosine triphosphate (ATP). ATP production is significantly more efficient in the presence of oxygen, with more ATP being produced in the conditions of aerobiosis [27].

During ischemia, less oxygen and substrates are delivered to cardiac myocytes, which will lead to the less aerobic production of ATP. As a consequence of energy depletion, anaerobic glycolysis is triggered, and lactate is produced. Although initially lactate is utilized for the production of ATP, albeit with less metabolic efficiency, with prolonged ischemia it accumulates in the cells, decreasing the intracellular pH, which will further inhibit the glycolytic enzymes and lead to further energy depletion, calcium cellular overload and finally to altered cellular function and death [27,28].

While acute prolonged ischemia causes irreversible cellular damage, if reperfusion is established in time, the dysfunctional myocardium called “stunned myocardium” can potentially recover its function in hours or days after the normal coronary flow has been established. In the “stunned” myocytes, contractility is downregulated by the decrease in the intracellular pH, with significantly less ATP needed, thus making possible myocyte viability in conditions of reduced oxygen supply. However, myocardial reperfusion can itself produce further cellular damage and the extension of the infarcted area. The establishment of normal perfusion leads to rapid intracellular pH normalization, which will further lead to mitochondrial “re-energization”, which will produce abundant ATP and reactive oxygen species (ROS). ROS damage the sarcoplasmic reticulum and cause a further increase in intracellular calcium. The result is cardiac myocyte hypercontracture and death. Furthermore, ROS act as chemoattractants for neutrophils, which further contribute to the local inflammation and damage [22,27,28].

During chronic ischemia, cardiac myocytes undergo metabolic and functional changes as an adaptive mechanism to decreased oxygen disponibility. The ischemic myocardium becomes hypocontractile, thus with a smaller consumption of ATP, being called “hibernating myocardium”. Although free fatty acids are normally the main source of ATP, the hibernating tissue manifests a shift towards glucose substrate, as a protective mechanism to enable anaerobic metabolism during ischemic episodes. Although in “hibernating myocardium” dysfunction is reversible after reperfusion, it can progress to irreversible cellular damage and myocardium necrosis in the case of prolonged, severe ischemia and in the absence of reperfusion, finally leading to adverse ventricular remodeling [27,28].

##### Renin–Angiotensin–Aldosterone System (RAAS)

RAAS activation after MI leads to elevated circulating and tissue levels of angiotensin II (Ang II). By stimulating angiotensin type 1 (AT1) receptors, Ang II causes potent vasoconstriction and sodium and water retention, increasing cardiac preload and afterload, and subsequently increasing the WS. Additionally, Ang II directly affects the myocardium by causing cardiomyocyte hypertrophy and the hyperplasia of cardiac fibroblasts, promoting fibrosis. Furthermore, it promotes ECM deposition and the release of other growth factors and mediators, such as norepinephrine and endothelin, which will contribute to cardiac remodeling [29,30]. Aldosterone plays a major role in the adverse remodeling after MI, as it regulates and promotes LV collagen deposition, cardiomyocyte apoptosis, endothelial dysfunction and fibrosis. It also regulates sodium and potassium plasmatic concentrations, and it further activates the RAAS through feedback mechanisms [31,32,33,34].

##### Sympathetic Nervous System (SNS)

The SNS is activated immediately following acute MI. In the early phase of remodeling, sympathetic overdrive promotes neutrophil influx in the necrotic area and infarct expansion by activating apoptotic pathways [35]. Subsequently, the stimulation of β-1-adrenergic receptors (β1-ARs) activates signaling pathways, which promote cardiomyocyte hypertrophy. In the juxtaglomerular apparatus, activated β1-ARs induce renin release, which enhances the Ang II production of β-2-adrenergic receptors (β2-ARs) that may have cardioprotective effects, such as anti-apoptotic properties [30].

##### Endothelin

Endothelin is a potent vasoconstrictor peptide that is stimulated by hypoxia, ischemia, neurohormones (norepinephrine, Ang II) and inflammatory cytokines. Endothelin 1 (ET-1) contributes to adverse ventricular remodeling as it promotes inflammation, due to its ability to activate macrophages, release inflammatory cytokines (Tumor necrosis factor-alpha- TNF-α, Interleukin 6- IL-6, Interleukin 1 beta- IL-1β), increase adhesion molecule expression and stimulate neutrophil aggregation [36,37]. Moreover, ET-1 stimulates cardiomyocyte hypertrophy [36,38].

##### Natriuretic Peptides

The natriuretic peptide (NP) system includes three structurally homologous peptides, A-type NP (ANP), B-type NP (BNP) and C-type NP (CNP), that are secreted by the atrial and ventricular cardiomyocytes due to the increase in WS and stretching of the peri-MI tissue [22]. The NPs promote diuresis, natriuresis, vasodilation, as well as the inhibition of RAAS, endothelin production and SNS activation [39]. ANP inhibits apoptosis and cardiomyocyte hypertrophy, as well as collagen synthesis, which is the main driver of cardiac fibrosis [40]. NP concentrations might be used to stratify patients at risk for remodeling [22,41,42,43,44,45].

##### Extracellular Collagen Matrix (ECM) Changes

The cardiac ECM is a highly organized network of structural and functional proteins that surrounds the cardiomyocytes and produces a cellular scaffold that maintains the LV shape and geometry [12,46,47]. The ECM proteins are degraded by matrix metalloproteinases (MMPs), which in turn are inactivated by tissue metalloproteinase inhibitors (TIMPs) [48,49]. After MI, ECM undergoes significant changes. Initially, during the local inflammatory response from the infarct area, neutrophils degrade cellular debris and release MMPs, which cause aberrant degradation of the ECM and can lead to infarct expansion. At around day 3 after the MI, neutrophils are followed by macrophages, which will promote fibroblast differentiation to myofibroblasts, causing the synthesis of large amounts of ECM to form the infarct scar [3,47,50,51,52]. At the same time, TIMPs are activated, leading to collagen accumulation and scar formation. After around 2 weeks, the scar begins to maturate, as the deposition of collagen increases and leukocytes and fibroblasts are cleared, most probably through apoptosis [53,54,55,56,57,58].

## 3. Biomarkers of Adverse Cardiac Remodeling

The role of biomarkers in the diagnosis, prediction, stratification and prevention of LV remodeling is not clearly stated. Several new biomarkers have been introduced in recent years, as summarized in Table 1.

### 3.1. Biomarkers of Cardiac Injury and Necrosis

The classical biomarkers of myocardial necrosis have recently been supplemented with several new biomarkers, including the heart-type fatty acid binding protein (hFABP), the ischemia-modified albumin (IMA) and the sarcomeric cardiac myosin-binding protein C (cMyC).

***hFABP*** is a low-molecular-weight, non-enzymatic protein involved in the intracellular buffering and transport of long-chain fatty acids. It is released into the circulation within an hour from the onset of the ischemic event, with levels returning to baseline in 12–24 h. Because it is relatively tissue-specific for the heart, hFABP has good specificity for myocardial necrosis [59,60]. Moreover, hFABP is a potential prognostic biomarker for long-term mortality. Matsumoto et al. showed that during the recovery stage, the hFABP levels in patients with prior MI could predict long-term mortality and the probability of readmission due to potential HF, even after discharge [61].

Acute myocardial ischemia causes significant protein changes, including alterations of the N-terminus of albumin, which lead to the formation of ***IMA***. IMA levels increase within 3 h following acute MI onset, but with reduced specificity for ischemia, since it also increases in a wide range of other medical conditions [62].

***Sarcomeric cMyC*** is a myosin-binding protein isoform expressed only in the heart and it is one of the most promising new myocardial necrosis biomarkers. It is a specific marker of myocardial injury that is released into the bloodstream more quickly than troponin, allowing the earlier detection of myocardial injury [63,64].

### 3.2. Inflammatory Biomarkers

***Inflammatory cytokines*,** such as TNF-α, IL-1β and IL-6 are significantly associated with LV adverse remodeling. These cytokines are released shortly after the ischemic injury and can acutely regulate myocyte survival or apoptosis, as well as initiate subsequent cellular inflammatory response [65,66]. Furthermore, cytokine secretion promotes gradual myocyte apoptosis or hypertrophy, and has effects on ECM by activating MMPs and collagen formation, mediating tissue repair and cardiac remodeling [16,67,68,69,70].

***Soluble Suppression of Tumorigenicity-2 (ST2)*** is a member of the IL-1 receptor family that is expressed on endotheliocytes and secreted by cardiomyocytes and fibroblasts when under mechanical stress. It has a membrane-bound form (ST2 ligand- ST2L), as well as a soluble form (soluble ST2-sST2). IL-33 is a functional ligand of the ST2L receptor. The binding of IL-33 and ST2L on the inflammatory cell membrane activates intracellular signaling pathways and triggers the inflammatory response. On the other hand, the binding of IL-33 and ST2L on the cardiomyocyte membrane has a protective effect against the Ang II adverse remodeling effects, preventing myocardial fibrosis and maladaptive hypertrophy. However, sST, which increases in MI, acts as a decoy receptor for IL-33, decreasing the levels of IL-33 available to interact with ST2L. Therefore, the cardioprotective effects of IL-33/ST2L are attenuated when the levels of sST2 are increased. The sST2 secretion is associated with increased myocardial fibrosis, LV remodeling and unfavorable cardiovascular outcomes after MI [71,72,73,74,75,76].

***Growth Differentiation Factor-15*** (GDF-15) is a member of the transforming growth factor-β (TGF-β) superfamily, and it is highly expressed in the myocardium and endothelial cells in patients with cardiovascular disease. GDF-15 is an inflammatory and oxidative stress biomarker and recent research suggests a possible role of GDF-15 in myocardial fibrosis, hypertrophy and endothelial dysfunction [77]. GDF-15 might be an independent marker of LV remodeling after MI and an integrative biomarker of HF in patients with acute MI [78,79].

***Myeloperoxidase*** (MPO) is an enzyme contained in neutrophils and monocytes, that is deployed in the extracellular environment by degranulation when inflammation is triggered. It is a promising, novel inflammatory biomarker as recent studies have shown that it is not only an excellent biomarker for diagnosing acute MI but also for identifying patients with vulnerable plaques, with an increased risk of plaque rupture [80,81,82,83].

### 3.3. Biomarkers of Cardiac Fibrosis

***Galectin-3*** is a β-galactoside-binding lectin that is mainly synthesized by macrophages from the infarcted tissue [84,85]. Apparently, in the early phase of MI, galectin-3 is involved in a reparative process in the infarcted area, which is critical for maintaining the LV geometry and function [86]. However, in the late phase of post-MI remodeling, galectin-3 stimulates tissue fibrosis and scar formation, therefore, being associated with cardiac remodeling. According to experimental studies, myocardial galectin-3 expression increases after MI, while several clinical studies indicate a relationship between elevated circulating levels of galectin-3 and a phenotype prone to HF after MI [16,87,88].

### 3.4. Biomarkers of Collagen Turnover

Excessive ECM protein production and accumulation, along with dysregulated turnover, promote post-MI remodeling and progression to HF. Targeting either protease-mediated fragmentation products (indicative of protein degradation) or the pro-peptide that is cleaved off the molecule during its maturation (indicative of protein formation) could offer information on ECM turnover. Circulating peptides released during collagen syntheses, such as ***carboxyterminal telopeptide of collagen type I*, *amino-terminal propeptide of type III procollagen***, as well as ***MMPs***, and ***TIMPs***, may be helpful for risk stratification of the remodeling process [47,89,90]. Using multiple biomarkers, including the traditional biomarkers and indicators of ECM turnover, may increase the sensitivity and specificity of clinical outcomes in patients with post-MI remodeling [16].

### 3.5. Biomarkers of Biomechanical Myocardial Stress

***NPs***, as previously mentioned, promote vasodilatation AND natriuresis and have antiproliferative and antifibrotic effects, preventing adverse ventricular remodeling [16,91,92]. Therefore, NPs are excellent prognostic biomarkers of remodeling after acute MI. According to the 2020 European Society of Cardiology (ESC) guidelines for the management of acute coronary syndromes in patients presenting without persistent ST-segment elevation, BNP and N-terminal pro-brain natriuretic peptide (NT-proBNP) plasma concentrations should be considered when assessing patient’s prognosis [93].

***Copeptin*** is a stable glycopeptide derived from the C-terminal fragment of the vasopressin prohormone, which is an essential regulator of water homeostasis and plasma osmolality, with increased secretion in conditions of stress. Copeptin is released in an equimolar ratio to arginine vasopressin within the first 4 h following an acute MI. Therefore, copeptin might be an excellent surrogate marker of arginine vasopressin secretion, which has a relatively short half-life. However, copeptin is not a specific cardiac marker, as the circulating levels are also influenced by other conditions, including kidney disease or sepsis [16,94,95,96].

***Midregional Proadrenomedullin*** (MR-ProADM) is a stable peptide fragment that serves as a precursor for adrenomedullin (ADM). ADM is a peptide hormone synthesized by the endothelial and vascular smooth muscle cells [16]. By binding to specific receptors, ADM increases nitric oxide and cyclic guanosine monophosphate synthesis, promoting vasodilation. It also mediates natriuresis, and it has a positive inotropic effect. Therefore, ADM exerts cardiac protection through several mechanisms: coronary vasodilation with subsequent increased myocardial blood flow and impaired maladaptive cardiac remodeling due to its antioxidant, antiapoptotic and antifibrotic effects [97]. In a study by Yoshitomi that evaluated plasmatic ADM in MI, the level of plasmatic ADM increased in the early stages of acute MI proportionately to the clinical severity, and it was further increased in patients with congestive HF [98]. In the LAMP Study, a significant increase in plasmatic MR-proADM after MI correlated with poor cardiac outcomes [99,100,101]. Furthermore, the increased levels of MR-proADM in acute MI patients, particularly in those developing HF, are associated with high rates of short and long-term mortality and hospitalization for HF [102,103].

### 3.6. Circulating Ribonucleic Acids

Non-coding ribonucleic acids (RNAs) are strong tissue- and cell-specific epigenetic regulators of cardiac gene expression and cell function. They are promising biomarkers in a broad spectrum of cardiovascular diseases and are widely investigated. ***MicroRNAs*** (miRNAs) are a class of tissue-specific or cell-specific small non-coding RNAs that regulate cell growth, proliferation, differentiation and apoptosis. Therefore, miRNA are extensively involved in cardiac remodeling in HF and after MI. Four miRNAs: ***miR-1*, *miR-133a/b*, *miR-208b*** and ***miR-499*** have increased circulating levels in acute MI, the last two being expressed exclusively in the cardiomyocytes [104,105]. MiRNAs are not only prognostic biomarkers in HF and ventricular remodeling but also targets for personalized intervention and translational therapy, with demonstrated effects in preventing maladaptive myocyte growth and improving ventricular function [22,106].

## 4. Imaging Techniques of Adverse Remodeling

Ventricular remodeling evaluation relies on assessing ventricular geometry and function, which can be performed with echocardiography or CMR (cardiac magnetic resonance).

***Two-dimensional (D) echocardiography*** is widely accessible, and it is the first imaging technique to be used when evaluating ventricular remodeling. Left ventricular volumes, ventricular contractility and left ventricular ejection fraction calculated by the biplane summation of disks method are the main parameters that characterize ventricular remodeling. It can also evaluate valvulopathies and their mechanism, such as tethering in secondary mitral regurgitation, which is indicative of adverse remodeling. However, ***3D echocardiography*** offers more reproducible information when assessing ventricular volumes and function, especially when using contrast for the best delineation of the endocardial borders. Furthermore, it provides supplementary information compared to 2D echocardiography when assessing valvulopathies. ***Strain echocardiography*** is a newer echocardiographic technique and it offers a more comprehensive evaluation of myocardial contractility, being able to detect the subclinical disease as well. In addition, it is an excellent tool to assess the prognosis of patients with HF [22,107].

***CMR*** is the gold standard imaging technique for the evaluation of ventricular volumes and function. However, the most valuable information derives from its ability to characterize the myocardial tissue. T2 mapping can identify myocardial edema, and, therefore, guide the diagnosis towards a cause of acute inflammation, such as acute myocarditis. Fibrosis is evaluated with T1 mapping, while the ventricular scar is assessed with late gadolinium enhancement imaging (LGE). Ventricular scar transmural extension determines the viability and subsequently guides potential coronary revascularization or cardiac resynchronization therapy (CRT). Scar localization offers further insight into the etiology of myocardial dysfunction, since it is mainly subendocardial in ischemic heart disease and mid-wall in dilated cardiomyopathy [108]. Furthermore, LGE can identify microvascular obstruction after MI, by visualizing the no-reflow phenomenon [22]. Finally, CMR is extremely useful in evaluating the etiology of MINOCA, and novel CMR techniques will bring further insight into ventricular remodeling after MI [17,22,107,108].

## 5. Therapeutic Implications in Adverse Cardiac Remodeling

The treatment in MI aims to prevent or attenuate left ventricular remodeling by reducing the size of the infarction and targeting the neurohormonal systems [12,13]. Reverse remodeling is the result of myocardial recovery in response to therapy, and it has been defined in previous studies as a decrease in LV end-systolic volume of 12% by CMR, while echocardiographic parameters include a reduction in end-systolic and end-diastolic volumes and an increase in LV EF of 12% to 15% [107,109]. It appears that in ischemic cardiomyopathy, there is no difference in the incidence of adverse remodeling between sexes. However, women have a lower predisposition to develop spherical distortion of the left ventricle, but a higher risk than men of developing HF [58]. In contrast, in non-ischemic cardiomyopathy, the rate of reverse remodeling is similar between sexes, but women have better outcomes [110]. The different response to therapy in adverse remodeling might be due to the fact that women develop less inflammation and apoptosis, with a less extended infarct area; sex hormones contribute to these processes [58]. Recent studies demonstrated that guideline-directed medical therapy, including angiotensin receptor neprilysin inhibitors promoting reverse remodeling both in ischemic and non-ischemic cardiomyopathy, improve the mortality and hospitalizations for HF. However, ischemic heart disease was associated with a lower probability of reverse remodeling and smaller improvements in ventricular diameters [111,112].

**Figure 3:**
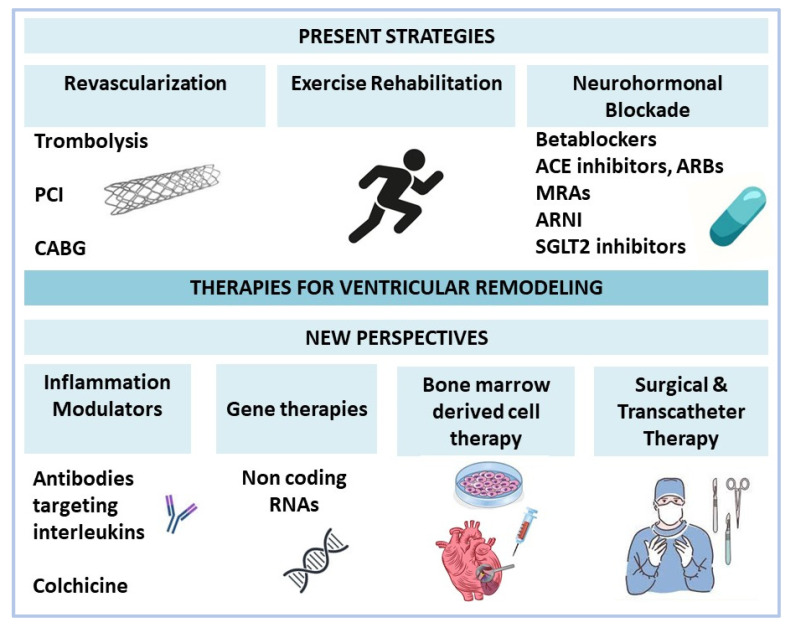
**Therapies for ventricular remodeling.** ACE: angiotensin-converting enzyme; ARBs: Ang II receptor blockers; MRAs: mineralocorticoid receptor antagonists; ARNI: angiotensin receptor neprilysin inhibitor; SGLT2: sodium-glucose cotransporter 2; RNAs: ribonucleic acids; PCI: percutaneous coronary intervention; CABG: coronary artery bypass graft.

The main therapeutic strategies to prevent or reduce adverse remodeling are summarized in Figure 3.

### 5.1. Myocardial Revascularization

The main goal of treatment in acute MI is to restore myocardial perfusion and prevent necrosis through thrombolysis, percutaneous coronary intervention (PCI) or coronary artery bypass graft (CABG) surgery, with a significant reduction in mortality. The benefit of revascularization on cardiac remodeling is the reduction in the size of the infarction, with the consequent improvement of regional and global ventricular function [13,113,114].

The restoration of coronary flow by PCI is the standard of care for patients presenting in the first 12 h from symptom onset. In a prospective observational study, Marek Grabka et al. demonstrated a high rate of reverse cardiac remodeling in individuals diagnosed with the first anterior-wall STEMI and treated with primary PCI [115]. In the absence of PCI, early thrombolysis should be considered, and various studies have shown that early onset thrombolysis has reduced the extent of ventricular wall kinetic abnormalities. Current European and American guidelines recommend starting fibrinolysis in up to 30 min from the first medical contact in all eligible patients with STEMI when PCI is not possible [12,116,117]. Despite the evolution and success rate of PCI myocardial revascularization, cardiac remodeling continues to occur in one-third of patients with AMI [118].

### 5.2. Exercise-Based Cardiac Rehabilitation

Exercise-based cardiac rehabilitation is recommended, in addition to drug therapy, after an MI in order to prevent the progression of cardiac remodeling. The beneficial cardiac effects are caused by a decrease in Ang II secretion, sympathetic activity and circulating catecholamine levels, as well as an improvement in the balance between MMP-1 and TIMP-1 [12,119]. These results in reverse remodeling, with a reduction in the LV diameters and an improvement in LV contractility. In addition, Zhang et al. showed that changes in left ventricular remodeling were more significant when exercise training programs were initiated in the acute phase after MI. According to the 2020 ESC Guidelines on sports cardiology and exercise in patients with cardiovascular disease, patients should be referred to an early exercise training program for 8–12 weeks after an acute coronary syndrome (ACS) in order to reduce cardiac mortality and rehospitalization [120,121,122].

### 5.3. Neurohormonal Inhibition

#### 5.3.1. Sympathetic Nervous System Blockade

SNS blockade plays a major role in the therapeutic strategy after MI. American and European guidelines support the use of beta-blockers after MI as a Class I recommendation. Long-term efficacy on morbidity and mortality was demonstrated by the CAPRICORN trial in which carvedilol was compared with a placebo in post-MI patients and with a LV EF ≤ 40% [123,124,125]. The benefit of beta-blockers is given by the inhibition of the effects of circulating catecholamines and reduction in heart rate and myocardial contractility, thus decreasing the oxygen demand. This prevents long-term interstitial fibrosis and significantly improves left ventricular remodeling [22,124,126,127,128]. In the CAPRICORN Echo substudy, carvedilol had a beneficial effect on ventricular remodeling: in the group of patients treated with carvedilol, there was a smaller increase in left ventricular end-systolic volume and a higher EF at 6 months after MI compared to controls [12,129]. In another study, Lee et al. compared the effects of propranolol and carvedilol on the volume and function of LV in patients benefiting from PCI after acute MI. Similar changes in LV end-diastolic volume in both groups were observed, proving that propranolol may be as beneficial as carvedilol [130,131].

#### 5.3.2. RAAS Blockade

RAAS blockade has clearly demonstrated, in the landmark trials, beneficial effects on mortality and cardiovascular events in patients with MI, especially with low EF.

**Angiotensin-converting enzyme inhibitors (ACEIs)** prevent the formation of Ang II, and, therefore, block all its deleterious effects, improve LV remodeling and ameliorate the progression to HF. Current guidelines include treatment with ACEIs as Class I recommendations, based on numerous clinical trials that demonstrated ACEIs benefits on mortality and left ventricular systolic function after acute MI [3,13,132,133,134]. Trials, such as ISIS-4, GISSI-3, CCS-1, CONSENSUS-2 and SMILE, focused on the administration of ACEIs in the first 24 h, while in more recent trials (TRACE, AIRE), treatment was initiated 48 h after the acute event. In all studies, mortality after MI was significantly reduced. A meta-analysis of all major studies supports the benefit of ACEIs in the early and later management of MI [135,136].

**Angiotensin II receptor blockers (ARBs)** are recommended as an alternative when patients do not tolerate or have contraindications to ACEIs. By blocking the AT1 receptors, ARBs improve sodium and water retention, and prevent cardiac hypertrophy and fibrosis, thereby improving post-infarction ventricular remodeling [3,137,138,139,140]. Studies have shown a similar efficacy of ARBs compared to ACEIs post-MI. The OPTIMAAL clinical trial compared the efficacy of captopril with losartan, while in the VALIANT study captopril was compared with valsartan. Both ARBs showed similar efficacy with captopril on mortality. Regarding ventricular remodeling, the VALIANT Echo substudy showed that treatment with captopril or valsartan resulted in similar changes in ventricular size and function after MI [12,22,141]. The advantage of ARBs compared to ACEIs is given by fewer adverse effects, thus having a better tolerability profile. Moreover, the absence of degradation of bradykinin by ARBs appears to have beneficial effects on the cardiovascular system, as demonstrated by current research [140,142].

**Mineralocorticoid receptor antagonists (MRAs)** are currently recommended in the treatment of STEMI in patients with ventricular systolic dysfunction (EF < 40%) and HF or diabetes, who are already on treatment with ACEIs and beta-blockers. This recommendation of ESC guidelines is based on the results of the EPHESUS study, which compared the administration of eplerenone vs. placebo in addition to standard therapy in post-MI patients with LV dysfunction and HF or diabetes. After an average follow-up of 16 months, a reduction in all-cause mortality, cardiovascular mortality and sudden cardiac death by 15%, 17% and 21%, respectively, was observed [12,137,143,144,145]. The REMINDER study evaluated the effect of MRAs initiated in the first 24 h in STEMI patients without a history of HF, with a composite primary endpoint of cardiovascular mortality, re-hospitalization or extended initial hospital stay, due to diagnosis of heart failure, sustained ventricular arrhythmias, EF < 40%, or elevated BNP/NT-proBNP at 1 month or more after randomization. After 13 months of follow-up, the results showed good tolerance of eplerenone and a reduction in the primary endpoint, mainly due to significantly decreased natriuretic peptide levels. In contrast, ALBATROSS trial included patients with STEMI and non-STEMI and assessed the benefit of early MRA initiation in acute MI in addition to standard therapy (versus standard therapy alone), regardless of the presence of HF or LV dysfunction. The primary outcome was a composite of death, resuscitated cardiac arrest, ventricular arrhythmias, indication for implantable defibrillator or new or worsening HF at 6 months follow-up. However, the ALBATROSS trial failed to show benefits from early MRAs administration in addition to standard therapy in patients with MI. Experts consider that both trials were underpowered individually to validly evaluate the hard endpoints. The discrepancies might arise also from the fact that the ALBATROSS trial included both STEMI and NSTEMI patients. Furthermore, a pooled analysis of the REMINDER group and the ALBATROSS STEMI subgroup showed that mortality was significantly lower in patients treated with MRAs versus placebo [137,146,147].

#### 5.3.3. Angiotensin Receptor Neprilysin Inhibitor (ARNI)

ARNI (sacubitril/valsartan) is a novel therapy that simultaneously inhibits the activation of RAAS by blocking AT1 receptors, and the degradation of bradykinin and natriuretic peptides by inhibiting neprilysin [148,149,150]. It is now considered one of the main pillars in the treatment of HF with reduced EF, since it demonstrated a significant reduction in mortality and hospitalization, greater than ACEIs, as shown by the PARADIGM–HF trial [151].

The effects of ARNI in patients with acute MI were studied in the PARADISE–MI study, which compared sacubitril/valsartan with ramipril in patients with acute MI and with an LV EF < 40% and/or signs of pulmonary congestion. The results of this study showed that sacubitril/valsartan did not reduce cardiovascular death or first HF hospitalization, compared to ramipril, however, it had a clear benefit in all HF events [152,153]. Moreover, in a recent meta-analysis, Zhang et al. showed that sacubitril/valsartan therapy after MI prevents adverse ventricular remodeling, thus improving cardiac function and reducing the rate of adverse cardiovascular events [149].

### 5.4. Sodium–Glucose Cotransporter 2 Inhibitors (SGLT2-I)

SGLT2-I, also called gliflozins (empagliflozin, canagliflozin and dapagliflozin), is another novel therapy that nowadays represents a cornerstone in the management of HF, given the outstanding results firstly showed by the landmark cardiovascular outcome trials EMPA-REG, CANVAS and DECLARE-TIMI, in which SGLT2-I significantly reduced cardiovascular mortality, all-cause mortality and HF hospitalization [154,155,156]. Empagliflozin and dapagliflozin reduced the incidence of recurrent MI, which may be related to the gliflozins capacity to reduce the ischemic-reperfusion injury [157,158].

In MI, SGLT2 inhibitors switch the myocardial substrate utilization from glucose towards ketone bodies, free fatty acids and branched-chain amino acids, thereby improving myocardial energetics. Experimental evidence shows that SGLT2 inhibitors exert cardioprotective effects in animal models with acute MI by improving cardiac function during ischemia, reducing infarction size and subsequently attenuating HF development [159]. The early initiation and continuation of SGLT2 inhibition after acute MI could be beneficial to prevent ventricular remodeling and progression to chronic HF [160,161,162,163,164,165,166,167]. Patients with acute MI have been relatively understudied in SGLT2 inhibitor outcome trials to date. Currently, there are three trials ongoing, EMPACT-MI, EMMY and DAPA-MI, which will evaluate the efficacy and safety of the early initiation of SGLT2 inhibitors within days of an acute MI [168].

### 5.5. Statins

The use of statins is recommended by international guidelines for the secondary prevention of cardiovascular and cerebrovascular events. New evidence suggests that statins also manifest beneficial effects against cardiac remodeling by inhibiting the proliferation of cardiac fibroblasts and ECM turnover. Experimental studies have shown that statins improved LV dilation after MI, thus limiting cardiac remodeling [137,169,170,171,172].

### 5.6. Inflammation Modulators

Since inflammation plays a major role in ventricular remodeling post-MI, several cytokines could be therapeutic targets in modulating myocardial inflammation. Blocking IL-1β signaling improves heart remodeling and the possible progression to HF. Canakinumab, a human monoclonal antibody that inhibits IL-1β, was evaluated in the CANTOS study. Canakinumab, compared to a placebo in patients with prior MI, increased C-reactive protein (CRP), reduced circulating CRP levels and decreased the incidence of recurrent cardiovascular events [3,173,174,175,176,177,178,179,180,181,182,183,184,185,186]. Several studies have shown that the inhibition in mice of the NLR family pyrin domain containing 3 (NLPR3), the macromolecule involved in regulating IL-1β and IL-18 activation, preserves cardiac systolic function following in vivo ischemic and non-ischemic damage. The administration of colchicine, a non-specific inhibitor of NLPR3, resulted in a significantly reduced infarct size in mice and improved cardiac function after acute MI [3,177,178,179,180].

### 5.7. Gene Therapy

Gene therapy is an emerging therapeutic tool with significant implications in adverse remodeling post-MI. Angiotensin-(1-9) is a novel peptide that regulates the RAAS. A study by Fattah et al. using in vivo gene transfer in a murine model of MI showed that Angiotensin-(1-9) gene therapy preserved LV systolic function after MI, restoring cardiac function [181]. Non-coding RNAs are major regulators of adverse remodeling after MI, in chronic HF and when the ventricular WS is increased. Silencing microRNAs in vivo using specific antisense inhibitors prevents maladaptive remodeling and improves cardiac function [182,183]. In a study by Danielson et al., extracellular plasma RNAs after MI were associated with phenotypes of left ventricular remodeling [183]. Another study found that up-regulation of miR-17 in diabetic mice improved left ventricular function after acute MI and reduced the size of the infarction [22,184,185,186].

### 5.8. Cell-Based Therapy in Cardiac Remodeling

Bone marrow-derived cell (BMC) therapy enables the repair of the damaged myocardial tissue after MI by promoting transdifferentiation of progenitor cells into healthy cardiomyocytes. The REPAIR–AMI trial evaluated the effect of intracoronary transplantation of BMCs on post-MI remodeling and showed that the intracoronary infusion of enriched BMCs is certainly linked with the improvement of the LV global function in patients with acute MI. At 12 months post-transplantation of BMCs, transplanted patients had a significantly reduced incidence of death, re-infarction and revascularization [22,187,188]. However, other studies showed neutral results of BMC therapy in patients with MI, and further research is needed [189,190,191].

### 5.9. Surgical and Transcatheter Interventions

Surgical procedures aimed to correct the distorted LV geometry with large areas of akinesia and aneurysms did not prove to be superior when compared with reperfusion therapy alone. Therefore, surgical procedures that restore the ventricular shape are indicated for selected HF patients who have refractory symptoms or malignant ventricular arrhythmias [22]. Minimally invasive transcatheter procedures can be performed in patients with chronic anteroseptal infarction, with the exclusion of the anterior scarred myocardium from viable tissue by plicating the anterior and LV free wall scar against the right ventricle septal scar [22]. This technique reduces the LV volume and restores conical LV morphology, potentially improving the LV systolic function [192].

## 6. Conclusions

LV adverse remodeling after MI is a process of major importance as it leads invariably to HF, increased mortality and a high economic burden. Understanding the pathophysiology and implementing the potential biomarkers that predict adverse remodeling make possible an integrative therapeutic approach and a better estimation of prognosis. Novel imaging techniques offer a more detailed evaluation of adverse remodeling, as well as the response to therapies that target the maladaptive process. Advances have been made in the therapeutic field, and timely coronary reperfusion in associations with novel therapies, such as ARNI and SGLT2-I, along with MRAs and beta blockers, counteract adverse ventricular remodeling and promote reverse ventricular remodeling, decreasing progression to HF and mortality. Future therapeutic perspectives, such as microRNAs, bone marrow derived-cells, and molecules targeting inflammation are currently under research, with promising results.

## Figures and Tables

**Table 1:** Biomarkers of adverse cardiac remodeling.

Biomarkers of cardiac injury and necrosis	hFABP IMA cMyC
Inflammatory biomarkers	TNF-α, IL-1β, IL-6 sST2 GDF-15 MPO
Biomarkers of cardiac fibrosis	Galectin-3
Biomarkers of collagen turnover	Carboxyterminal telopeptide of collagen type I Amino-terminal propeptide of type III procollagen MMPs TIMPs
Biomarkers of biomechanical myocardial stress	BNP, NT-proBNP Copeptin MR-proADM
Circulating ribonucleic acids	miR-1 miR-133a/b miR-208b miR-499

hFABP: heart-type fatty acid binding protein; IMA: ischemia-modified albumin; cMyC: sarcomeric cardiac myosin-binding protein C; TNF-α: tumor necrosis factor alpha; IL-1β: interleukin 1 beta; IL-6: interleukin 6; sST2: soluble suppression of tumorigenicity-2; GDF-15: growth differentiation factor-15; MPO: myeloperoxidase; MMPs: matrix metalloproteinases; TIMPs: tissue metalloproteinase inhibitors; BNP: B-type natriuretic peptide; NT-proBNP: N-terminal pro-brain natriuretic peptide; MR-proADM: mid-regional proadrenomedullin.

## Data Availability

Not applicable.

## Abbreviations

MI: myocardial infarction; HF: heart failure; LV: left ventricle; RAAS: renin-angiotensin-aldosterone system; SNS: sympathetic nervous system; ECM: extracellular collagen matrix; SV: stroke volume; WS: wall stress; ATP: adenosine triphosphate; ROS: reactive oxygen species; Ang II: angiotensin II; AT1: angiotensin type 1 receptor; β1-ARs: β-1-adrenergic receptors; β2-ARs: β-2-adrenergic receptors; ET-1: endothelin 1; TNF-α: tumor necrosis factor alpha; IL-6: interleukin 6; IL-1β: interleukin 1 beta; NP: natriuretic peptide; ANP: A-type natriuretic peptide; BNP: B-type natriuretic peptide; CNP: C-type natriuretic peptide; MMPs: matrix metalloproteinases; TIMPs: tissue metalloproteinase inhibitors; hFABP: heart-type fatty acid binding protein; IMA: ischemia-modified albumin; cMyC: sarcomeric cardiac myosin-binding protein C; sST2: soluble suppression of tumorigenicity-2; GDF-15: growth differentiation factor-15; TGF-β: transforming growth factor-β; MPO: myeloperoxidase; ESC: European Society of Cardiology; NT-proBNP: N-terminal pro-brain natriuretic peptide; MR-proADM: mid-regional proadrenomedullin; ADM: adrenomedullin; RNAs: ribonucleic acids; miRNAs: microRNAs; CMR: cardiac magnetic resonance; LGE: late gadolinium enhancement; CRT: cardiac resynchronization therapy; PCI: percutaneous coronary intervention; CABG: coronary artery bypass graft; ACS: acute coronary syndrome; EF: ejection fraction; ACEIs: angiotensin-converting enzyme inhibitors; ARBs: Ang II receptor blockers; MRAs: mineralocorticoid receptor antagonists; ARNI: angiotensin receptor neprilysin inhibitor; SGLT2: sodium-glucose cotransporter 2; T2D: type 2 diabetes; CRP: C-reactive protein; NLPR3: NLR family pyrin domain containing 3; BMC: bone marrow-derived cell.
